# Effects of glucosamine against morphine-induced antinociceptive tolerance and dependence in mice

**DOI:** 10.1186/s12929-019-0513-1

**Published:** 2019-02-19

**Authors:** Faezeh Basiri, Abolfazl Rad, Davood Mahdian, Mehdi Molavi, Bahareh Amin

**Affiliations:** 1 Student Research Committee, Sabzevar University of Medical Sciences, Sabzevar, Iran; 2 Cellular and Molecular Research Center, Department of Physiology and Pharmacology, Faculty of Medicine, Sabzevar University of Medical Sciences, Sabzevar, Iran; 3 Departement of Internal Medicine, Faculty of Medicine, Sabzevar University of Medical Sciences, Sabzevar, Iran

**Keywords:** Morphine, Dependence, Tolerance, Glucosamine, Mice

## Abstract

**Background:**

The most important limitations of morphine in pain therapy are its tolerance and dependence. In this study, we evaluated the protective effect of glucosamine against morphine-induced tolerance and dependence in mice.

**Methods:**

Mice received twice daily morphine (20 mg/kg, s.c.) alone, or along with orally administered glucosamine (500, 1000 and 2000 mg/kg), for 9 continuous days. To assess antinociceptive effect of morphine, percentage of maximal possible effect (%MPE) of animals exposed to thermal stimulus was measured in the hot plate test, 30 min after morphine administration. Test was performed on days 1, 3, 5, 7 and 9. The effect of glucosamine on the naloxone (5 mg/kg, i.p.)-precipitated morphine withdrawal, was also evaluated. Changes in brain gene expression levels of induced nitric oxide synthase (iNOS), enzyme responsible for nitric oxide generation, as well as pro-inflammatory mediator, tumor necrosis alpha (TNF-α) were measured in morphine tolerated animals, as well as after withdrawal by real-time polymerase chain reaction (RT-PCR). Protein content of TNF-α was evaluated via ELISA assay.

**Results:**

Tolerance to antinociceptive effect of morphine was developed after 7 days of morphine treatment. The concurrent administration of glucosamine (500, 1000 and 2000 mg/kg) with morphine, significantly inhibited tolerance development, on days 7 and 9. In addition, glucosamine ameliorated the naloxone-precipitated opioid withdrawal symptoms (tremor, jumping, teeth chattering, grooming). However, diarrhea was significantly improved only with the dose of 500 mg/kg. Increased mRNA expression of iNOS as well as TNF-α mRNA expression and protein, after both morphine tolerance and withdrawal, were considerably reduced by glucosamine (1000 mg/kg) in the morphine withdrawal animals.

**Conclusion:**

These data support the utility of glucosamine in attenuating both tolerance to nociceptive effects of morphine as well as withdrawal-induced behavioral profile. Anti-oxidant and anti-inflammatory effects are responsible, at least in part, for the protective effects of this drug.

## Introduction

Opioids (narcotics) including morphine have been used to relief acute and chronic kinds of pain, and rank among the most powerful analgesic drugs. However, side effects especially tolerance to analgesic effects in patients chronically treaded with such drugs, are common [1, 2]. Another problem by opioids is physical dependence with withdrawal avoidance behaviors, limiting their therapeutic utility in clinic. This situation occurs when opioids are suddenly ceased or an antagonist is taken by patients. The main symptoms including insomnia, agitation, nausea, vomiting and diarrhea are appeared soon after withdrawal. To overcome opioids tolerance and dependence, we have to increase the dose of drugs and give unceasing drug regimen which leads to induce other side effects of morphine [1].

After chronic administration of morphine, microglia and astroglia (glial) cells tend to be activated. Many cytokines including tumor necrosis factor-α (TNF-α), as a pro-inflammatory cytokine, nitric oxide (NO), free radicals, prostaglandins, neurotrophic factors and excitatory amino acids are released. All of these factors mediate neuronal activation and CNS sensitization, affecting morphine efficacy [3–5].

Dietary supplement glucosamine sulfate (2-amino-2-deoxy-D -glucose; GlcN), is widely used to increase joint comfort with inducing formation of structural proteo-glycans in joint cartilage, which could be a good option in treatment of arthritis [6].

Glucosamine has been reported to have anti-tumor [7], anti-oxidant [8, 9], and anti-allergic activity [10]. Other pharmacological properties of GlcN including protective effects against multiple sclerosis and encephalomyelitis [11], learning and memory impairment [12], colitis [13], and ischemic brain injury [14], have been investigated.

The present study was performed to evaluate the protective effects of GlcN against tolerance to analgesic effect of morphine as well as withdrawal reactions precipitated by morphine antagonist, naloxone. To find possible mechanisms of actions contribute in tolerance to morphine as well as naloxone-induced withdrawal, inducible nitric oxide synthase (iNOS), enzyme responsible for the generation of nitric oxide (NO), responsible for reactive nitrogen species (RNS) production, and pro-inflammatory mediator, tumor necrosis factor-α (TNF-α), were measured by real-time polymerase chain reaction (RT-PCR). Protein content of TNF-α were also measured through Enzyme-linked immunosorbent assay (ELISA).

## Materials and methods

Morphine hydrochloride and naloxone were purchased from Darupakhsh Co., (Iran). Glucosamine sulfate was obtained from Sigma Chemical Co., (USA). All drugs were dissolved in normal saline.

### Animals

Male albino *Swiss* mice (*n* = 54), 3–4 weeks of age, weighing 30–35 g, were housed in a pathogen-free cages on a 12-h light/dark cycle and fed with standard laboratory diet and tap water ad libitum under controlled temperature (23 ± 2 °C). Prior to the experiments, animals were provided adaptive feeding for 7 days. All procedures were done between 8 and 13 AM. Animals care and handling procedures were followed in according to the National Institute of Health Guide for the Care and Use of Laboratory Animals [15]. All applied procedures were approved by the Animal Care and Use Committee of the Sabzevar University of Medical Sciences, Sabzevar, Iran (ir.medsab.rec.v1394.126).

### Study design

To induce tolerance, mice were administered morphine (20 mg/kg), subcutaneously (s.c.), twice a day for 9 days, based on the previous study [16]. Animals in the treated groups also received glucosamine with gavage, twice a day, 30 min before morphine administration during the days of study.

The development of tolerance to analgesic effect of morphine was evaluated by the hot plate test, 30 min after morphine administration, on days 1, 3, 5, 7 and 9. Mice were randomly allocated to one of the nine groups:

1) Morphine treated group (*n* = 6) received morphine (20 mg/kg), twice a day, plus vehicle at 12 h intervals for 9 days.

2) Control group (*n* = 6) received normal saline, twice a day for 9 days.

3) Glucosamine group (*n* = 6) was treated with 1000 mg/kg of glucosamine alone, twice a day for 9 days.

4–6) Testing groups A (*n* = 6) received glucosamine (500, 1000, 2000 mg/kg) via gavage, twice daily, 30 min before each morphine injection (20 mg/kg) twice a day, for 9 days.

7–9) Testing groups B (*n* = 6) received glucosamine (500, 1000, 2000 mg/kg) via gavage, twice daily, 30 min before each morphine injection (20 mg/kg) twice a day, for 9 days and also naloxone (5 mg/kg, i.p), 2 h after the last administration of morphine.

The selected doses were according to the previous data in the literature [14].

#### Behavioral tests

##### Assessment of antinociceptive effect of morphine

Animals were placed into a Plexiglas cylinder (24 cm diameter, 30 cm height) fixed on the heated surface of hot plate (Borj Sanat, Iran). The time between placing of the animal on the hot-plate and the occurrence of licking of hind paws or jumping off the surface was recorded as the response latency. One day before test, animals were first habituated to the apparatus. An automatic 28 s was considered as the cut-off time, to prevent tissue damage [17]. Data were expressed as a percentage of maximal possible effect (%MPE) according to the following equation:

Drug latency-Basal latency/Cut off latency-Basal latency×100.

##### Assessment of withdrawal symptoms

Abstinence-like syndrome was evaluated by a single administration of antagonist, naloxone (5 mg/kg, i.p.), 2 h after the last dose of morphine for groups 1, 2, 4–6. Withdrawal signs were characterized by the duration of tremor (sec), number of jumping, number of teeth chattering, number of grooming and number of defecation as an indicator of diarrhea, for 30 min as described previously [18].

#### RNA extraction and qRT-PCR

After detecting anti-nociceptive effect of morphine in testing groups A and withdrawal manifestations in testing groups B, animals were decapitated. Brain tissues were immediately removed from mice treated nine days with intraperitoneal normal saline, morphine alone, morphine+ glucosamine and morphine+glucosamine which received a single dose of naloxone on the last day of treatment. The tissues were immediately frozen in the liquid nitrogen and stored at − 80 °C until usage for extraction of total RNA (write half of brain) and protein (left half of brain).

RT-PCR was performed with total RNA extracted from the homogenized sample (approximately, 1 mg tissue), using Tri Pure Isolation Reagent (Roche Diagnostics, Deutschland GmbH, Germany). To avoid DNA contamination, extracted RNAs were treated with RNase-free DNase I (Thermo Fischer scientific, USA) followed by heat inactivation in the presence of EDTA. Total RNA (2 μg) was converted into cDNA using the PrimeScript™ RT reagent Kit (Takara Bio Inc., Japan) according to the manufacturer’s instructions. Real- time PCR was carried out using SYBR green PCR master mix (YTA, Iran) on CFX96 Touch™ Real-Time PCR Detection System (Bio-Rad, Philadelphia, PA USA), under following thermal conditions: 94 °C for 10 min, 94 °C for 15 s at 39 cycles, 57 °C for 20 s, 72 °C for 30 s. The PCR Primer sequences are presented in Table 1. The data were normalized by glyceraldehyde 3-phosphate dehydrogenase (GAPDH) expression, using comparative threshold cycle method [19]. Each reaction was performed in triplicates.

**Table 1:** Primers specific for rat inducible nitric oxide synthase (iNOS), and tumor necrosis alpha (TNF-α) and Glyceraldehyde-3-Phosphate Dehydrogenase (GAPDH), forward and reverse. F: Forward primer sequence. R: Reverse primer sequence

1	F(iNOS, Rattus)	GGGTCTTGTTAGCCTAGTCA
2	R(iNOS, Rattus)	TGTTGTTGGGCTGGGAATAG
3	F(TNFα, Rattus)	CCCAACAAGGAGGAGAAGTT
4	R(TNFα, Rattus)	GGCTTGTCACTCGAGTTTTG
5	F(GAPDH, Rattus)	AGCTCATTTCCTGGTATGACA
6	R (GAPDH, Rattus)	TTGCTCTCAGTATCCTTGCT

#### ELISA analysis

At the time of experiment, samples were thawed at room temperature and homogenized using a tissue homogenizer (Heidolph, Germany) in a homogenization buffer consisting 20 mmol/LTris (pH 7.4; Sigma-Aldrich), 150 mmol/L NaCl (Sigma-Aldrich), 1 mmol/L ethylene diamine tetraacetic acid (Sigma-Aldrich), 2 mmol/L 2-N-morpholinoethanesulfonic acid or 2 ME (Sigma-Aldrich), protease inhibitor (Sigma-Aldrich) (Reece et al., 2004). Total protein contents were determined by the Bradford assay and adjusted [20]. Cytokine levels was measured by the commercial available ELISA kit specific for TNF-α (Diaclone, France). Each sample was evaluated triplicates. Analysis of protein content was performed according to the manufacturer’s instructions from the standard curve.

#### Statistical analysis

Data were shown as mean ± SEM and analyzed with SPSS version 19 software (Chicago: SPSS Inc.). Statistical analyses were carried out by mixed model ANOVA with repeated measure and post hoc bonferroni for evaluating antinociceptive activity. Withdrawal and RT-PCR data were analyzed by one way ANOVA followed by the Tukey post hoc test for multiple comparisons. The *p*-values less than 0.05 were considered to be statistically significant.

## Results

### Effect of glucosamine on the development of morphine tolerance

It should be noted that glucosamine alone (1000 mg/kg) treated mice didn’t show a significant difference with normal saline control ones, indicating no significant analgesic effect.

For percentage of MPE a 6 (time) *6 (treated) mixed model ANOVA revealed significant main effects of time (F (5, 150) = 45, *p* < 0.001), treatment (F (5, 30) = 18.3, p < 0.001), as well as significant time*treatment interaction (F (25, 150) = 5.8, *p* < 0.001).

%MPE for morphine treated group revealed a significant increase on days 1, 3 and 5 compared to control group (*p* < 0.01). However, %MPE was significantly decreased in morphine treated group compare to control one from day 7, which remained until day 9.

**Fig. 1:**
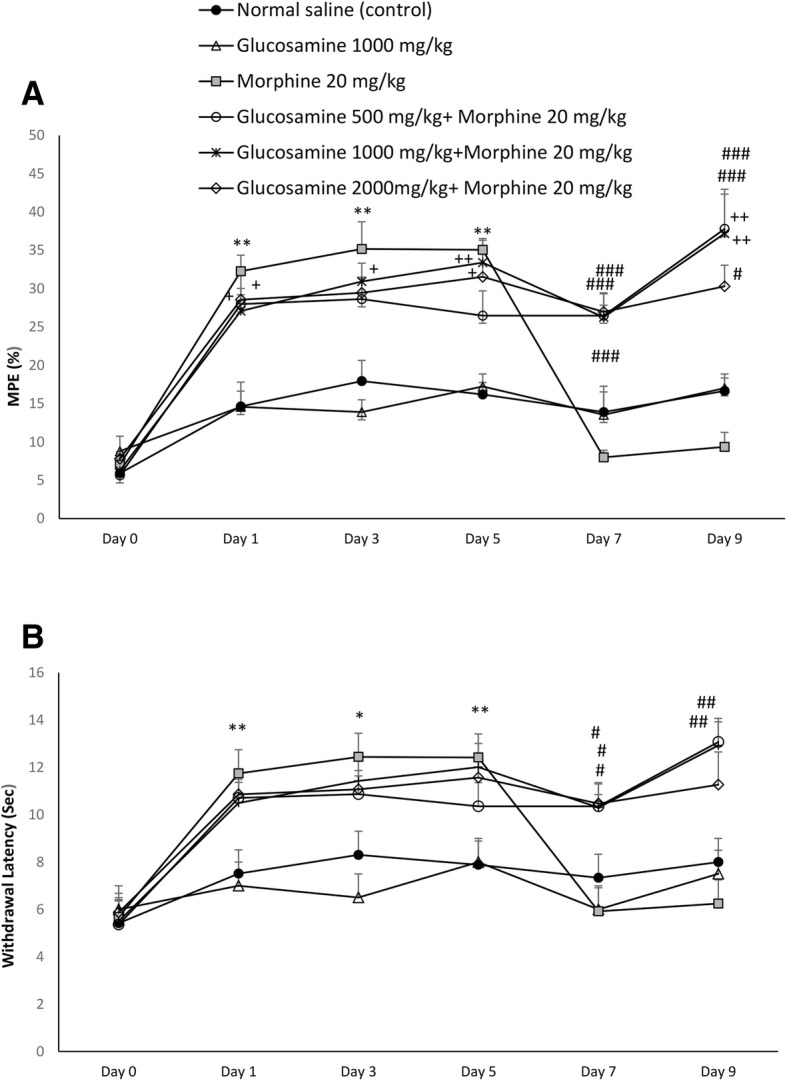
Time course effect of glucosamine on the development of tolerance to morphine (20 mg/kg, s.c., twice daily), during 9 days in mice. **a**: maximum possible effect (%), **b**: Paw withdrawal latency. Glucosamine was administered via gavage (500, 1000 and 2000 mg/kg, twice daily, for 9 days). Analgesia was measured 30 min after the treatment, on the hot plate apparatus. Values are expressed as mean ± SEM (*n* = 6). Statistical analyses was performed by mixed model ANOVA with repeated measure and post hoc bonferroni for evaluating antinociceptive activity. *p < 0.05, **p < 0.01 comparison between morphine and control (normal saline) groups. #*p* < 0.05, ##*p* < 0.01, ###*p* < 0.001 treated glucosamine+morphine as compared to morphine treated group. +*p* < 0.05, ++*p* < 0.01, treated glucosamine+morphine as compared to control (normal saline) group

In glucosamine+morphine treated groups (500, 1000, 2000 mg/kg), %MPE was significantly maintained on days 7 and 9 as compared to morphine treated group (Fig. 1a).

This improvement was as much as there was a significant difference between the dose of 500 mg/kg and control normal saline, regarding the %MPE on day 9 (*p* < 0.05). There was also a significant difference between the dose of 1000 mg/kg and normal saline on days 1 (*p* < 0.05), 5 (*p* < 0.01), and 9 (*p* < 0.01). A significant difference was also observed between the dose of 2000 mg/kg and normal saline on days 1 (*p* < 0.05), 3 (*p* < 0.05) and 5 (*p* < 0.05) (Fig. 1a).

The 6 (time) *6 (treated) mixed model ANOVA revealed significant main effects of time (F (5, 150) = 29.7, *p* < 0.001), treatment (F (5, 30) = 7.8, *p* < 0.01), as well as significant time*treatment interaction (F (25, 150) = 5.5, *p* < 0.001) for withdrawal latency.

Withdrawal latency for morphine treated group revealed a significant increase on days 1 (*p* < 0.01), 3 (*p* < 0.05) and 5 (*p* < 0.01) compared to normal saline control group. In morphine treated group latency to thermal stimulus was significantly decreased from day 7, which showed no significant difference in relation to control group (*p* > 0.05). This effect remained until day 9.

As shown in Fig. 1B, glucosamine significantly prevented withdrawal latency reduction on days 7 (with all doses, *p* < 0.05), and 9 (*p* < 0.01 for 500 and 1000 mg/kg, *p* < 0.05 for 2000 mg/kg) in comparison to morphine treated group.

### Effect of glucosamine on the development of morphine dependence

**Fig. 2:**
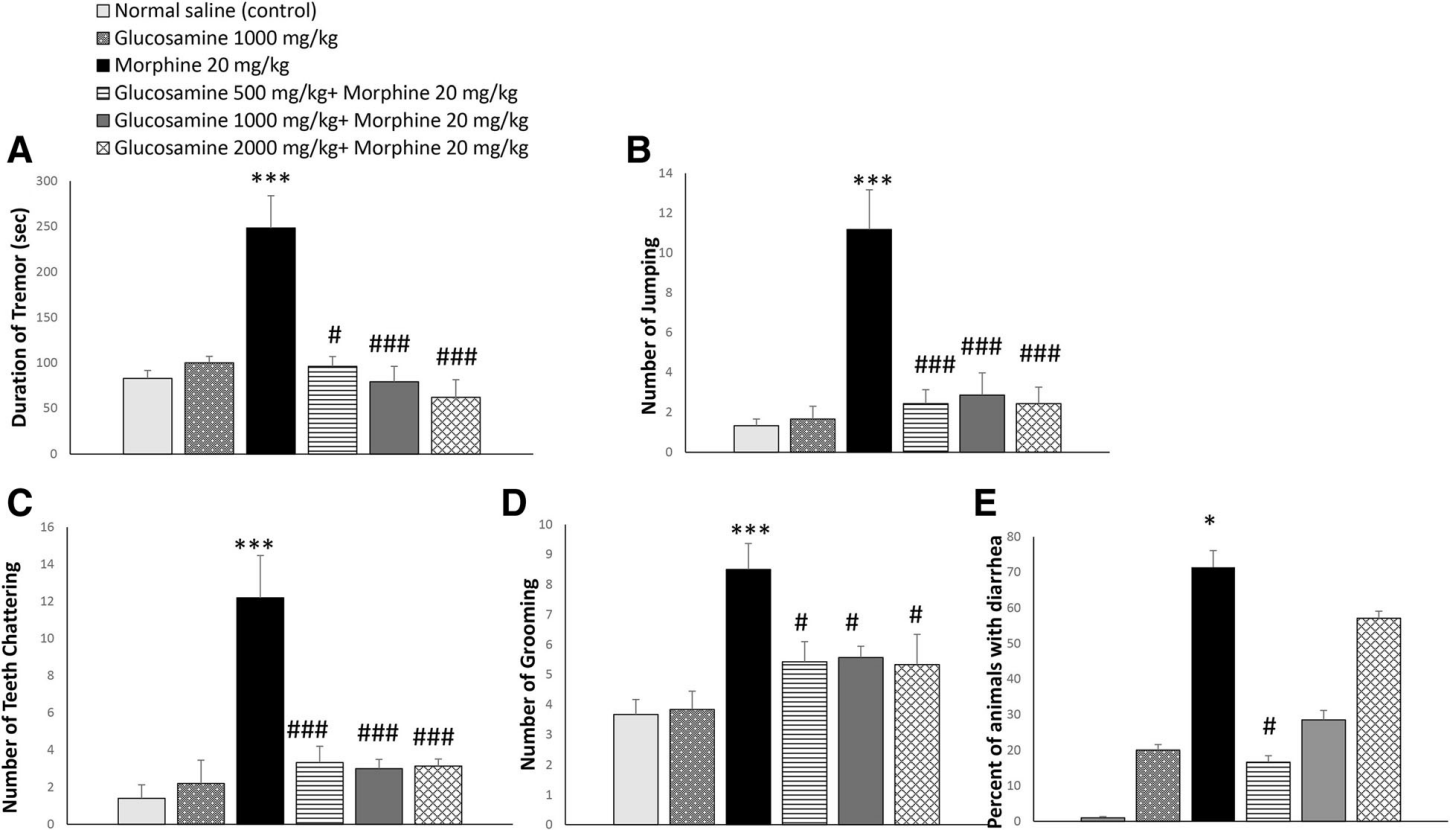
Effect of glucosamine on the development of naloxone (5 mg/kg, i.p.)-induced morphine withdrawal symptoms, 2 h after last injection of morphine. Glucosamine was administered via gavage (500, 1000 and 2000 mg/kg, twice daily, for 9 days). **a**: Duration of tremor; **b**: Number of jumping; **c**: Number of teeth chattering; **d**: Number of grooming; **e**: Percent of animals with diarrhea. Values are expressed as means ± SEM. One way ANOVA and post hock sidak was used. *p < 0.05, ***p < 0.001 as compared to control group; #*p* < 0.05, and ###*p* < 0.001 as compared to morphine treated group

Withdrawal manifestations induced by the antagonist, naloxone, such as number of paw tremor (F 5, 37 = 14.36; *p* < 0.001), number of jumping (F 5, 37 = 12.87; *p* < 0.001), number of teeth chattering (F 5, 35 = 9.6, *p* < 0.01) and number of grooming (F 5, 37 = 10.38; *p* < 0.001), were significantly increased in morphine-treated group compare to control group (Fig. 2a, b, c and d, respectively). Using Fisher exact test the number of animals with diarrhea significantly increased in morphine-treated group as compared to control group (*p* < 0.01; Fig. 2E).

In evaluation of tremor, glucosamine significantly reduced duration of tremor compared to morphine treated group in a dose dependent manner (*p* < 0.05 for dose of 500 mg/kg and *p* < 0.001 for doses of 1000 and 2000 mg/kg; Fig. 2a). Glucosamine significantly reduced number of jumping and number of teeth chattering compared to morphine treated group (*p* < 0.001, Fig. 2b and c). Number of grooming in glucosamine treated group was significantly lower than morphine treated group (*p* < 0.01 for 500 mg/kg and *p* < 0.001 for 1000 and 2000 mg/kg).

Glucosamine with dose of 500 mg/kg but not higher doses of 1000 and 2000 mg/kg significantly reduced the number of animals with diarrhea (*p* < 0.05; Fig. 2e).

### Effect of glucosamine on the iNOS mRNA expression in morphine tolerated animals and after morphine withdrawal

**Fig. 3:**
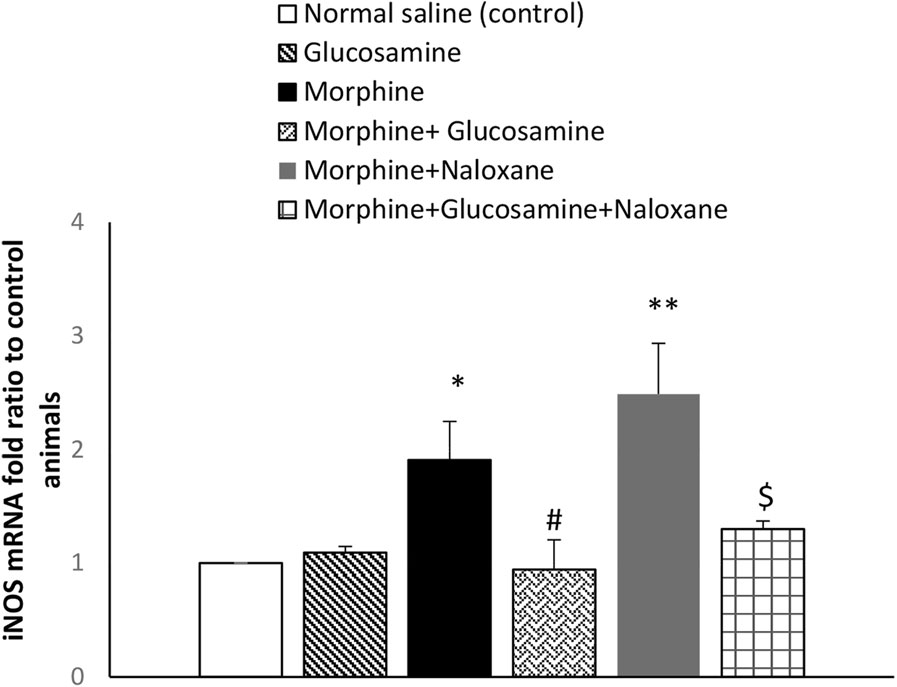
Effect of gavage glucosamine (1000 mg/kg, twice daily, for 9 days), on the mRNA levels of iNOS (induced nitric oxide synthase) in both morphine tolerated mice and morphine-dependent mice received naloxone (5 mg/kg, i.p.) challenge. GAPDH (glyceraldehyde 3-phosphate dehydrogenase) is a reference gene and was used for normalization as an internal control. Results (mean ± SEM) are expressed in terms of relative quantification (fold-change) using the 2^-ΔΔCT^ method for *n* = 4. One way ANOVA and post hock Tukey was used *p < 0.05, ***p < 0.001 vs. normal saline control group; #*p* < 0.05 morphine+glucosamine vs. morphine treated group. $*p* < 0.05 morphine+glucosamine+naloxane vs. morphine+naloxane treated group

We selected the effective dose of 1000 mg/kg of glucosamine for molecular examination. One way ANOVA showed a significant difference among groups in tolerated animals. F (3, 15) = 4.5, *p* < 0.05. Glucosamine alone did not affect the expression of iNOS. iNOS mRNA expression was significantly increased in the brain of morphine treated group compared to normal saline control group (*p* < 0.05) (Fig. 3). Increased iNOS mRNA expression in morphine-treated mice were reduced significantly by glucosamine 1000 mg/kg (*p* < 0.05).

A significant difference was detected among withdrawn animals groups with F (3, 15) = 9.34, *P* < 0.01. iNOS mRNA expression was significantly increased in the brain of abstinent animals from morphine compared to normal saline control group (*p* < 0.01). Increased iNOS mRNA expression in morphine-treated mice were reduced significantly by glucosamine 1000 mg/kg (*p* < 0.05; Fig. 3).

### Effect of glucosamine on the TNF-α mRNA expression and protein level in morphine tolerated animals and after morphine withdrawal

Evaluating tolerated animals, one way ANOVA showed a significant difference on the expression of TNF-α among morphine tolerated animals with F (3, 15) = 14.2, *p* < 0.001. Glucosamine alone did not affect the expression of TNF-α.

**Fig. 4:**
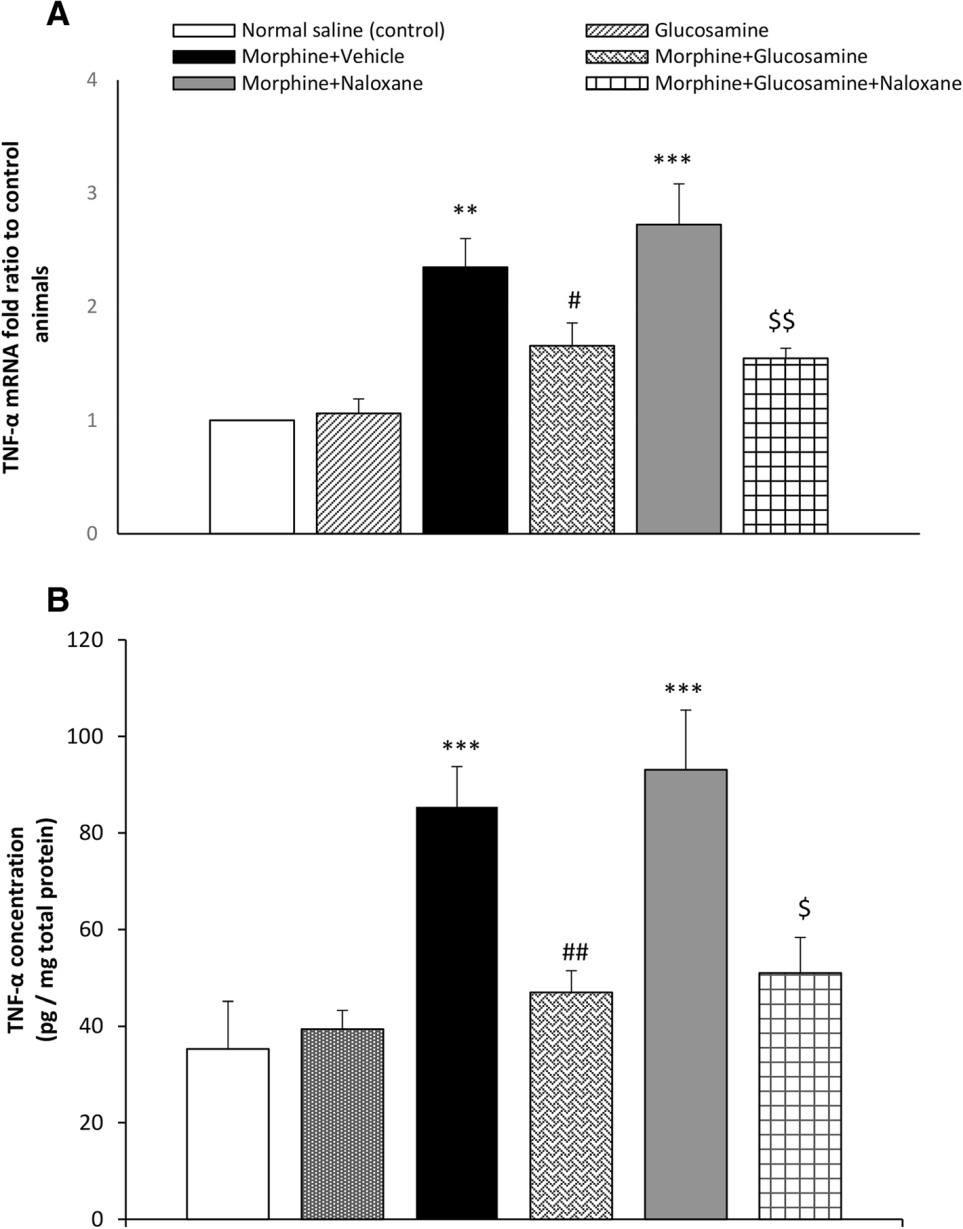
Effect of gavage glucosamine (1000 mg/kg, twice daily, for 9 days), on the A: mRNA levels of the TNF-α (tumor necrosis alpha) in both morphine tolerated and morphine-dependent mice received naloxone (5 mg/kg, i.p.) challenge. B: Protein levels of the TNF-α in morphine tolerated and morphine-dependent mice received naloxone (5 mg/kg, i.p.) challenge by ELISA assay. GAPDH (glyceraldehyde 3-phosphate dehydrogenase) is a reference gene and was used for normalization as an internal control. Results (mean ± SEM) are expressed in terms of relative quantification (fold-change) using the 2^-ΔΔCT^ method for *n* = 5. One way ANOVA and post hock Tukey was used. **p < 0.01, ***p < 0.001 vs. normal saline control group; #*p* < 0.05, ##*p* < 0.01 morphine+glucosamine vs. morphine treated group. $*p* < 0.05, $$*p* < 0.01 morphine+glucosamine+naloxane vs. morphine+naloxane treated group

The expression of mRNA specific for TNF-α, was significantly increased in morphine tolerated mice (*p* < 0.01), by comparison to normal saline controls. However, the mRNA expression levels of TNF-α showed a significant reduction in the brain samples of mice treated with morphine+ glucosamine 1000 mg/kg as compared to morphine treated group (*p* < 0.05) (Fig. 4A).

One way ANOVA showed a significant difference among withdrawn animals from morphine with F (3, 15) = 16.5, *p* < 0.001. The expression of mRNA specific for TNF-α, was significantly increased in morphine withdrawn animals (*p* < 0.001), by comparison to normal saline controls. However, the mRNA expression levels of TNF-α reduced in the brain samples of mice treated with morphine plus glucosamine 1000 mg/kg as compared to morphine treated group (*p* < 0.01) (Fig. 4a).

A significant difference among morphine tolerated groups was observed with F (3, 19) = 13.1, *p* < 0.00. Tolerance to morphine resulted in a significant increase in the protein brain levels of the proinflammatory cytokine, TNF-α as compared to normal saline control group (*p* < 0.001). Treatment with glucosamine, once daily, for nine days exhibited a significant decrease in the levels of TNF-α (Fig. 4b, *p* < 0.01).

One way ANOVA showed a significant difference among groups, F (3, 19) = 13.1, *p* < 0.001, among morphine withdrawal animals. Morphine withdrawal resulted in a significant increase in the brain levels, TNF-α as compared to normal saline control group (*p* < 0.001). Treatment with glucosamine, once daily, for nine days exhibited a significant decrease in the levels of TNF-α (Fig. 4B, *p* < 0.01).

## Discussion

Opioids, especially morphine, are the most widely used drugs in the treatment of acute and chronic pain, such as cancerous pain. Pharmacological tolerance is one of the most common side effects of morphine application, which is defined as a decrease in its effect with prolonged administration of a constant dose [21]. In addition to the morphine tolerance, development of withdrawal syndrome after abrupt ceasing of opioids is a prominent problem in patients [21]. In this study tolerance to antinociceptive effect of morphine was produced at the dose of 20 mg/kg/s.c., twice daily, started from day 7 of study. Glucosamine alone did not have any anti-nociceptive activity per se. Repeated administration of glucosamine with doses of 500, 1000 and 2000 mg/kg, 30 min before morphine administration, via oral route, retained antinociceptive effectiveness produced by morphine. This result demonstrates the attenuation to morphine-induced tolerance after 7 days of administration. However, best outcome was obtained with the dose of 1000 mg/kg of glucosamine.

We selected gavage administration of drug that is the most preferred, safe and readily available route of drug prescription with the most compliance of patients [22].

Based on earlier studies, potential pharmacokinetic interaction of glucosamine and morphine is excluded [23].

Morphine antagonist, naloxone (5 mg/kg, i.p.), induced withdrawal signs such as tremor, jumping, teeth chattering, increased grooming behavior and diarrhea on day 9 of morphine administration, indicating manifestation of physical dependence to morphine. However, abstinent animals received glucosamine (500, 1000 and 2000 mg/kg) concurrently with morphine, exhibited less withdrawal adverse effects, which was not dose dependent.

Although significant weight loss was not detected among group, withdrawal induced-diarrhea was not significantly reduced with the higher doses of 1000 and 2000 mg/kg. It could be explained by this fact that glucosamine itself, can cause some side effects at the higher doses including nausea, heartburn, diarrhea, and constipation [24].

Recent researches have suggested that release of large amounts of pro-inflammatory cytokines including TNF-α, in the CNS occur following chronic administration of morphine [25]. Such changes might have an important role in the central sensitization and thereby opioid-induced tolerance, as well as unwanted behavioral manifestations after withdrawal. TNF-α plays a significant role in the modulation of excitatory neurotransmitter, glutamate transmission and thereby interaction by opioid receptors [26]. Selective TNF-α inhibitor, etanercept, restored the antinociceptive effect of morphine in tolerated mice [27].

It has been proposed that the activation of μ-opioid receptors by opioids somehow initiates and/or increases the activation of glutamate receptors, particularly N-methyl D-aspartate (NMDA) receptors and following that increased glutamate release, NO and proinflammatory cytokines such as TNF-α. Activation of theses pathways lead to the desensitization of opioid receptors and consequently reduction in the antinociceptive activity of opioids [28–30].

Although NO could have neuroprotective effect, the role of NO, as a neuroexcitatory cytokine in the induction of morphine tolerance and dependence has been shown in previous studies [31, 32]. Substances can act as the inhibitors of producer enzyme, iNOS, reduce great amounts of NO, further oxidation and thereby morphine tolerance and dependence [33, 34]. Mice treated with iNOS inhibitor, aminoguanidine, elicited less withdrawal behaviors score [35]. On the other hand, GSH content as an antioxidant enzyme combating oxidative damage, is reduced after morphine administration and withdrawal of it [36, 37].

Our results showed that the mRNA expression of iNOS, TNF-α, as well as its protein content increased both in tolerated animals and after naloxone challenge in mice treated with 9 days morphine. However, glucosamine at the dose of 1000 mg/kg, was able to reverse such changes in both tolerance to morphine and naloxone-induced withdrawal syndrome.

Antioxidant activity of glucosamine has been attributed to its radical scavenging activity [38]. Hwang et al. showed that glucosamine decreased infarct volume in the post-ischemic brain rats. It also suppressed lipopolysaccharide (LPS)-induced inflammatory molecules such as prostaglandin E2, and interlukine 1. The authors found that glucosamine acted through inhibiting the activity of nuclear factor kappa B, a transcription factor, responsible for transactivation of the iNOS gene [14]. Such protective effects were not seen with similar compounds such as galactosamine, mannosamine and N-Acetylglucosamine. A novel glucosamine was also reported to have anti-inflammation against LPS [39]. Inflammation after ischemic stroke was also reduced by glucosamine in normotensive and hypertensive rats [40].

A recent study tested that glucosamine through inhibiting the enzyme responsible for metabolizing O-linked β-N-acetylglucosamine (O-Glc-NAcylation), could attenuate hyperexcitability of neurons in brain. This protein dampens neuronal synaptic strength in the hippocampus of the brain [41].

Inhibition of Ca^2+^ influx and outward K^+^ currents by glucosamine could inhibit microglia activation that offer potential use of this drug in treatment of inflammatory and neurodegenerative diseases [42].

In addition, glucosamine showed an excellent antioxidant and anti-inflammatory effects in mice exposed to cigarettes for 4 weeks [43]. It has been shown that glucosamine has a chelating tendency with ions such as ferrous that protects macromolecules such as protein, lipid, and deoxyribose from oxidative damage induced by hydroxyl radicals [44].

A limitation of our study was the lack of evaluation of astroglia and microglia activation markers to find the better results of glial activation during morphine-induced tolerance period. Measurement of these factors could complete the results of the study.

## Conclusion

Regarding that safety of glucosamine has been evaluated in previous studies [45], and prescription of this drug as an supplement is common in clinic, it could be a promising option in combination with morphine to retain its analgesic effect. Moreover, it seems that there is a close relationship between mechanisms contribute to the morphine tolerance and dependence. Anti-oxidative property, radical scavenging activity and anti-inflammation in the CNS could mediate the beneficial effects of glucosamine in both, tolerance and dependence upon chronic morphine administration. However, defining exact mechanisms of glucosamine protection require further explorations.

## Abbreviations

ELISA: Enzyme-linked immunosorbent assay
GlcN: Glucosamine sulfate (2-amino-2-deoxy-D –glucose)
iNOS: Inducible nitric oxide synthase
LPS: Lipopolysaccharide
NO: Nitric oxide
RT-PCR: Real-time polymerase chain reaction
TNF-α: Tumor necrosis factor-α
